# mapMECFS: a portal to enhance data discovery across biological disciplines and collaborative sites

**DOI:** 10.1186/s12967-021-03127-3

**Published:** 2021-11-08

**Authors:** Ravi Mathur, Megan U. Carnes, Alexander Harding, Amy Moore, Ian Thomas, Alex Giarrocco, Michael Long, Marcia Underwood, Christopher Townsend, Roman Ruiz-Esparza, Quinn Barnette, Linda Morris Brown, Matthew Schu

**Affiliations:** 1 Biostatistics and Epidemiology Division, RTI International, Research Triangle Park, NC USA; 2 Center for Data Science, RTI International, Research Triangle Park, NC USA

**Keywords:** Myalgic encephalomyelitis, Chronic fatigue syndrome, Data Sharing Portal, Comprehensive Knowledge Archive Network (CKAN), Data integration, Multi-omics

## Abstract

**Background:**

Myalgic encephalomyelitis/chronic fatigue syndrome (ME/CFS) is a debilitating disease which involves multiple body systems (e.g., immune, nervous, digestive, circulatory) and research domains (e.g., immunology, metabolomics, the gut microbiome, genomics, neurology). Despite several decades of research, there are no established ME/CFS biomarkers available to diagnose and treat ME/CFS. Sharing data and integrating findings across these domains is essential to advance understanding of this complex disease by revealing diagnostic biomarkers and facilitating discovery of novel effective therapies.

**Methods:**

The National Institutes of Health funded the development of a data sharing portal to support collaborative efforts among an initial group of three funded research centers. This was subsequently expanded to include the global ME/CFS research community. Using the open-source comprehensive knowledge archive network (CKAN) framework as the base, the ME/CFS Data Management and Coordinating Center developed an online portal with metadata collection, smart search capabilities, and domain-agnostic data integration to support data findability and reusability while reducing the barriers to sustainable data sharing.

**Results:**

We designed the mapMECFS data portal to facilitate data sharing and integration by allowing ME/CFS researchers to browse, share, compare, and download molecular datasets from within one data repository. At the time of publication, mapMECFS contains data curated from public data repositories, peer-reviewed publications, and current ME/CFS Research Network members.

**Conclusions:**

mapMECFS is a disease-specific data portal to improve data sharing and collaboration among ME/CFS researchers around the world. mapMECFS is accessible to the broader research community with registration. Further development is ongoing to include novel systems biology and data integration methods.

**Supplementary Information:**

The online version contains supplementary material available at 10.1186/s12967-021-03127-3.

## Background

Myalgic encephalomyelitis/chronic fatigue syndrome (ME/CFS) is a complex, debilitating disease [1] estimated to affect as many as 2.5 million Americans [2]. Affected individuals often have incapacitating fatigue, nonrefreshing sleep or other sleep difficulties, and cognitive impairment that may leave them unable to leave the house or bed. The disease is characterized by the worsening of symptoms following even minor physical or mental exertion, known as post-exertional malaise [3]. Although the underlying disease etiology remains unknown [1, 4] there is evidence that multiple body systems are involved. When comparing ME/CFS cases to controls, researchers have observed differences in the immune system [5, 6], blood metabolites [7–12], the gut microbiome [13–15], and mitochondrial DNA genetic variants [16]. Integrating findings across these domains promises to reveal a more complete picture of the disease, thereby detecting diagnostic biomarkers and facilitating discovery of novel effective therapies.

To support data sharing, the ME/CFS Research Network [17] comprising three Collaborative Research Centers and a Data Management and Coordinating Center (DMCC) was funded in 2017 by multiple National Institutes of Health (NIH) Institutes, Offices, and Centers, including the National Institute of Neurological Disorders and Stroke and the National Institute of Allergy and Infectious Diseases to encourage collaborative research to lead to better diagnosis and treatment for ME/CFS. One of the goals of the DMCC is to help ME/CFS researchers discover new disease insights by promoting data sharing. To this end, we developed the mapMECFS [18] data portal, which is built on a flexible database structure and optimized to handle multiple data types (e.g., gene expression, methylation, metabolomics, cytokine measures, proteomics, microbiome, survey/questionnaire). This portal contains specialized features to support cross-disciplinary and cross-study ME/CFS research generated from multiple research domains including cascading forms to collect key study metadata, intuitive dataset filtering, and smart search capabilities with synonym tagging with embedded mapping of synonymous feature terminologies.

## Methods

### Website framework and infrastructure

mapMECFS is built on the comprehensive knowledge archive network (CKAN) framework, an open-source tool designed to support data storage and sharing [19]. The portal includes an ecosystem of custom plugins hosted on a novel computing infrastructure built on Amazon Web Services technologies [16]. CKAN [20] includes a customizable containerization schema using Docker [21] in conjunction with Amazon Web Services [22] technologies to engineer a more performant, resilient, and affordable solution. Docker containers are minimal computing environments that can run on a personal computer, a dedicated server, or cloud computing. Docker containers are created from Docker Images, container definitions, that allow the portal developers to define the computing environment, network connections, and source code needed for each component of the portal. These images can be tested locally and then pushed to the cloud computing instance and activated as containers running the most up-to-date version of the software in a stable and predictable fashion. mapMECFS’ database components are hosted using Amazon’s Relational Database Service Aurora [23] database-as-a-service platform, which only incurs cost during times of usage and automatically scales to the size required by the contents of the database. User-uploaded files are stored using Amazon’s Elastic File System [24], a scalable and managed file system. By using these two services, we minimize cost of infrastructure and maintenance, as both services scale to usage for size and require no maintenance or management.

For the computation needed to run the application, mapMECFS utilizes Amazon’s Elastic Container Service (ECS) [25] running on top of Elastic Compute Cloud (EC2) [24]. mapMECFS’ EC2 compute server functions as a provisioned virtual machine with adequate and scalable resources for handling loads from both web traffic and more resource-intensive asynchronous tasks such as our custom extensions. ECS allows developers to dynamically allocate resources throughout the containerized computation services, including the web server, a Redis cache for managing queues, and an instance of Apache Solr [26] used for building search indexes. Additionally, mapMECFS leverages Amazon’s Cloudfront [27] content delivery network and Route53 [28] domain name service to maintain availability across the world wide web.

### mapMECFS access and site organization

mapMECFS is accessible to the research community at https://www.mapmecfs.org [18]. To obtain full access to the mapMECFS portal, new users must register for an account, provide a brief description of how they will use the data on the portal, and agree to the data use agreement. Account registration must be approved by the NIH data access committee. Upon approval, a user account will be created granting the user permission to explore and upload data.

mapMECFS site users are grouped together within an Organization which designates the institute, research center, or individual research lab that users and the datasets (a collection of data files) they upload are associated with (Additional file 1: Figure S1). A user must be part of an Organization to upload data. Independent or unaffiliated users are assigned to an Organization where they are the only member. Datasets are designated as either public (available to all mapMECFS site users) or private (only available to users within an Organization). Each user account has a defined role within an Organization. Organization members can view private datasets within an Organization on a read-only basis. Organization editors have all the abilities of members in addition to the ability to create and edit datasets they created and request a private dataset to be made public.

## Results

**Fig. 1:**
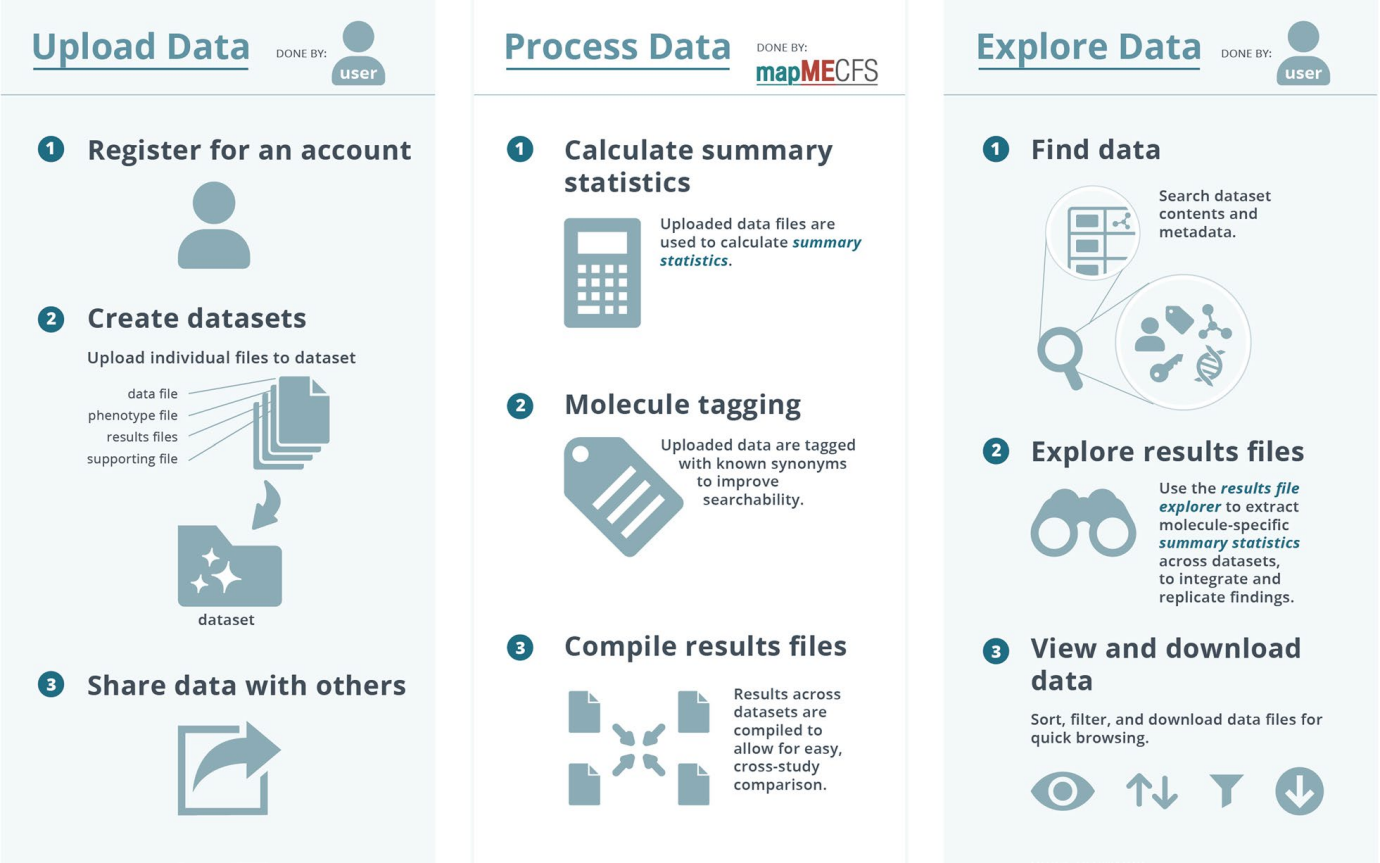
mapMECFS website overview. The user uploads files (data, phenotype, results, and supporting files) to a Dataset along with metadata. All datafiles are access protected to public or private depending on the user’s preference. mapMECFS processes the data to generate summary statistics, conduct synonym tagging, and compile results files to compare findings across datasets. The user can search available data and results files along with viewing, filtering, or downloading files

We created mapMECFS to facilitate sharing of ME/CFS data among the broader research community. To expedite that process, we populated the portal with current publications and publicly available data. An overview of the portal is shown in Fig. 1. Registered site users can upload their own de-identified primary research data and research results and share with other approved site users by creating a dataset and uploading associated files.

### mapMECFS data and curation

mapMECFS was designed to store de-identified demographic, survey, and health data coupled with molecular data (e.g., transcriptomics, metabolomics, methylation). The DMCC has curated ME/CFS data with open-access publications [5–9, 11, 13–16, 29–38]; gene expression, methylation, and micro-RNA (miRNA) datasets from the Gene Expression Omnibus [19, 39–48]; and metabolomic data from MetaboLights [49]. Active curation by the DMCC is ongoing (Additional file 1: Table S1).

Metadata, such as dataset title, description, tags, cohort selection, and case definition, are captured during the upload process for all datasets. The dynamic upload process prompts users for other key metadata based on the data type. Thus, metadata prompts for gene expression datasets will be different than metadata prompts for DNA methylation experiments. Metadata collection is performed using prepopulated, easy-to-use, drop-down menus to describe the experimental assay and data measurement unit with supplemental open-ended text boxes. Uploaded data are categorized by tags, which are suggested by natural language processing in real time based on a description provided by the user. Users can select the most appropriate suggested tags or supply their own as free text. These tags are used to filter and sort datasets to enable easy findability.

Data are uploaded into datasets. The definition of a dataset is flexible; it can contain one or many relevant file types (data file, phenotype file, results file, supporting files), as described in Table 1. To take advantage of the custom tools described below, the data, phenotype, and results files must follow the specified file format requirements described in Additional file 2: Figure S2. A dataset can contain an unlimited number of results and supporting files, thus enabling the sharing of standard operating procedures, external links (e.g., publication, sequencing read archive, data availability), and results from two or more analyses. The data upload process is flexible, allowing for multiple file formats. We encourage researchers to share datasets with other mapMECFS site users by making their datasets Public (i.e., viewable to all approved mapMECFS users). The process of approving public data requests will be conducted in accordance with the mapMECFS policies (“Methods” section).

**Table 1:** File types uploaded to mapMECFS, including data file (e.g., processed data), phenotype file (e.g., clinical data), results file (e.g., summary statistics), and support file (e.g., link to publication)

File type	Description	Examples
Data file	Processed data containing sample-level values as columns and molecules as rows. The header for each column should match the participant ID in the phenotype file. Only one data file can be uploaded per dataset	Gene expression counts Methylation signal intensities Metabolomics mass spectrometry peak heights
Phenotype file	Subject-level clinical values with participant ID matching that in the data file. Only one phenotype file can be uploaded per dataset	Case–control status Age Sex Relevant covariates
Results file	Summary statistics and other analysis output generated by the user, with statistics reported for each molecule	Wilcoxon-rank sum summary statistics with p-values and adjusted p-values
Supporting file	Additional documentation of experimental procedures or supporting material. Supporting documentation is recommended to provide users with a better understanding of the experiment generating the dataset	Standard operating procedures describing the dataset generation in more detail Hyperlinks to publications using the data included in the dataset Data dictionary

### Custom analysis and search tools

We optimized the mapMECFS search functionality to maximize data discovery and created custom analysis and search tools that run automatically once data is uploaded. The search feature recognizes user-specified terms describing multiple aspects of a dataset, including a dataset name, descriptions, key metadata, data file contents, and data synonyms generated from the *Synonym Tagging* tool [50] (Additional file 1: Table S2). *Synonym Tagging* is an automated backend feature of mapMECFS that labels molecules with known synonyms to enhance the searchability of analytes on the portal. By tagging both the indicated annotation and all recognized synonyms, mapMECFS extends the search space for each entered query. Synonyms are assigned based on well-established databases: National Center for Biotechnology Information’s Entrez [51] for gene expression data; InChIKey [52], ChEBI [53] ID, and HMDB [54] ID for metabolomic data; miRBase [55] for miRNA data; and manifest files for methylation Illumina array data (Illumina, Inc., San Diego, CA). For transparency, the results of the *Synonym Tagging* are available on the dataset page (Additional file 3: Figure S3) via the 'View Additional Search Terms' hyperlink. For example, if a researcher is interested in the cytokine interleukin 17, searches of “IL-17” or “IL-17A” will return all datasets containing the desired molecule, effectively standardizing terminology from different research domains and ontologies.

If the uploaded dataset contains both a data file with sample-level molecule values and a phenotype file with participant-level variables (e.g., case–control status), the *Calculated Summary Statistics* tool [56] will calculate and display a set of summary statistics for each molecule compared for each phenotypic group (e.g., case vs. control, multiple subtypes). These two-group comparison tests are performed using the nonparametric Wilcoxon Rank Sum test [57]. The *Calculated Summary Statistics* tool generates (1) sample sizes in each group, (2) median value for each group, (3) standard deviation for each group, (4) Wilcoxon rank-sum test statistics, (4) Wilcoxon rank-sum p-value, and (5) Wilcoxon rank-sum Bonferroni Corrected p-value, allowing researchers to quickly to characterize how analytes may differ between phenotype groups.

mapMECFS also includes a customized data search tool, the *Results File Explorer*, to facilitate cross-dataset searches. This tool is designed to allow users to quickly evaluate the reproducibility of a given result across multiple studies by comparing results to other analyses or subset analyses. Additional file 4: Figure S4 shows an example search for the metabolite 4-hydroxyglutamate. This amino acid, part of the glutamate metabolism pathway, has substantial implications in brain function [7] and has been shown to be elevated in ME/CFS patients [7]. The *Results File Explorer* output shown in Additional file 4: Figure S4 contain three separate tables, “Data Files and Calculated Summary Statistics”, “Results Files”, and “Other Datasets”. “Data Files and Calculated Summary Statistics” contains search results only from the mapMECFS *Calculated Summary Statistics*. The “Results Files” table contains search results only from the user-uploaded results files. The “Other Datasets” table contains search results from other elements of the dataset, including the title, description, or metadata. The tables contain key metadata so researchers can view analytic results while comparing analysis endpoints and applied methods. With the *Results File Explorer* tool and the mapMECFS search functionality, users can identify datasets to validate their findings and identify datasets to integrate with data they have collected. This process enhances the collaborative nature of ME/CFS research.

## Discussion

To promote international sharing of ME/CFS data across researchers and repositories, we created mapMECFS, a ME/CFS domain-specific data repository that conforms to NIH’s Findable, Accessible, Interoperable, and Reusable (FAIR) [58]. Guiding Principles and its strategic plan for data science [59]. mapMECFS achieves this by (1) being findable with persistent identifiers for each table within a dataset (and the dataset itself) and providing rich metadata, (2) making all data and metadata accessible via both an intuitive user interface and application programming interface, (3) providing data and metadata that are interoperable with vocabulary and language which are common in the field, and (4) using clear reuse license and data provenance. The mapMECFS portal is developed with a scalable infrastructure that delivers scientific impact to the research community, supports good data management practices, and actively engages the user community.

mapMECFS use cases may include (1) identifying new collaborators by identifying researchers working on a specific data type or by browsing descriptions of individual researchers’ interests, (2) validating a research finding by searching for a molecule of interest (in the Dataset explorer or *Results File Explorer*) to discover independent datasets containing that molecule, (3) meeting a funder or journal’s requirements of data sharing by uploading new experimental data or summary statistics, and (4) increasing statistical power by identifying an appropriate dataset for meta-analysis or data integration using the captured metadata, dataset description, and other support documentation. The DMCC plans to improve data integration by standardizing sample identifiers and clinical data capture to support systems biology and data integration approaches for ME/CFS research. Investigating multi-omics may help with identification for disease subtyping, diagnosis, and predictive outcome [60].

## Conclusions

mapMECFS [18] is available to the broad research community with registration as described above. To facilitate sharing of MECFS data, we encourage new users to consider mapMECFS for their research needs. This unique, domain-specific data repository was designed considering NIH’s FAIR Guiding Principles and strategic plan for data science. We encourage mapMECFS users to provide feedback and to request new features. mapMECFS is continuously expanding the types of data included on the site and welcomes feedback on the types of data researchers would like to share.

## Supplementary Information

**Additional file 1: Figure S1 and Table S1 and S2.** Regarding mapMECFS’ authentication organizational structure, publicly curated datasets, and synonym tagging.

**Additional file 2: Figure S2.** mapMECFS expected formats for (a) data (e.g., Cytokine data), (b) phenotype, (c) results files, and (d) the summary statistics file format.

**Additional file 3: Figure S3.** Dataset page for mapMECFS shows the metadata included for the dataset on the left. Each of the uploaded files is shown by the title, description, and file type. Options are available to preview, download, and edit each file. mapMECFS generated files are shown directly below the uploaded dataset files.

**Additional file 4: Figure S4**. Description: Results File Explorer example with a search for 4-hydroxyglutamate. The Results File Explorer shows three tables, “Data Files” and “Calculated Summary States,” “Results File,” and “Other Datasets.”

## Data Availability

The mapMECFS data portal is open to the research community at https://www.mapmecfs.org/. Code for the custom analyses and tools available in mapMECFS is available at https://github.com/search?p=1&q=user%3ARTIInternational+ckan&type=Repositories.

## Abbreviations

ChEBI: Chemical Entities of Biological Interest
CKAN: Comprehensive knowledge archive network
DMCC: Data Management and Coordinating Center
FAIR Principles: Findable, Accessible, Interoperable, Reproducible Principles
HMDB: Human Metabolomics Database
InChIKey: International Chemical Identifier Key
ME/CFS: Myalgic encephalomyelitis/chronic fatigue syndrome
miRNA: Micro-RNA
NIH: National Institutes of Health
