# Supplementary material for: mapMECFS: a portal to enhance data discovery across biological disciplines and collaborative sites
Source: J Transl Med. 2021 Nov 8;19:461. doi: 10.1186/s12967-021-03127-3 (PMC8576927; doi:10.1186/s12967-021-03127-3)
Supplement: Supplementary file 1 — Additional file 1: Figure S1 and Table S1 and S2. Regarding mapMECFS’ authentication organizational structure, publicly curated datasets, and synonym tagging. [file 12967_2021_3127_MOESM1_ESM.docx]

mapMECFS: a portal to enhance data discovery across biological disciplines and collaborative sites

Ravi Mathur*^1^, Megan Carnes*^1^, Alexander Harding^2^, Amy Moore^1^, Ian Thomas^2^, Alex Giarrocco^2^, Michael Long^2^, Marcia Underwood^2^, Christopher Townsend^2^, Roman Ruiz-Esparza^2^, Quinn Barnette^1^, Linda Morris Brown^1^, Matthew Schu^‡1^

^1^Biostatistics and Epidemiology Division, RTI International, Research Triangle Park, NC, USA

^2^Center for Data Science, RTI International, Research Triangle Park, NC, USA

*Co-first Authors

^‡^Corresponding Author – [mschu@rti.org](mailto:mschu@rti.org)

**Additional File 1**

**Figure S1:** *Schematic outlining the organizational structure of the mapMECFS portal, highlighting dataset privacy within Organizations.*


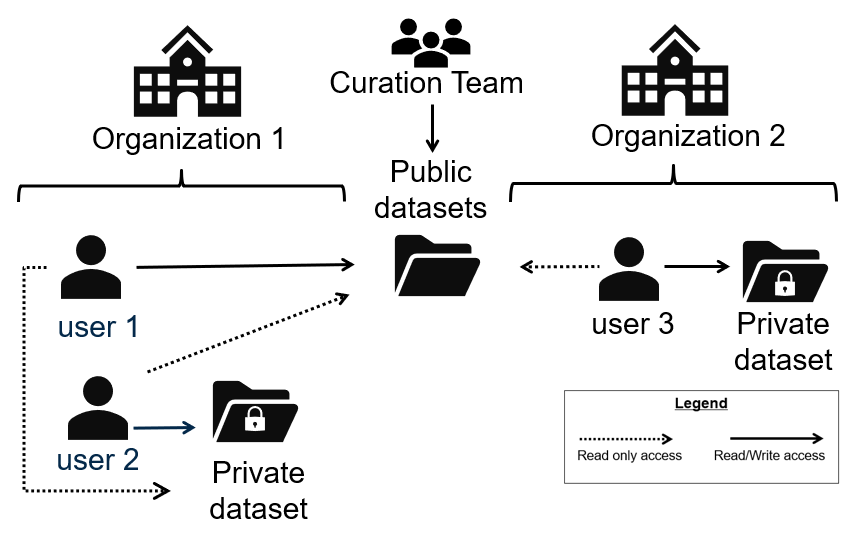


**Table S1:** *Public datasets curated into mapMECFS.*

| **Publication** | **mapMECFS Data Type** | **Source*** |
| --- | --- | --- |
| Giloteaux et al.^1, 2^ | Cytokine Assay | MECFSnet Publication |
| Hornig et al.^3^ | Cytokine Assay | MECFSnet Publication |
| Hornig et al.^4^ | Cytokine Assay | MECFSnet Publication |
| Montoya et al.^5^ | Cytokine Assay | Publication |
| Bouquet et al.^6^ | Gene Expression | GEO |
| Byrnes et al.^7^ | Gene Expression | GEO |
| Raijmakers et al.^8^ | Gene Expression | GEO |
| Gow et al.^9^ | Gene Expression | GEO |
| Armstrong et al.^10^ | Metabolomics | MetaboLights |
| Germain et al.^11^ | Metabolomics | MECFSnet Publication |
| Germain et al.^12^ | Metabolomics | MECFSnet Publication |
| Nagy-Szakal et al.^13^ | Metabolomics | MECFSnet Publication |
| Germain et al.^14^ | Metabolomics and Lipidomics | MECFSnet Publication |
| Kitami et al.^15^ | Metabolomics | Publication |
| Baraniuk et al.^16^ | Metabolomics | Publication |
| De Vega et al.^17-19^ | Methylation | GEO |
| Helliwell et al.^20^ | Methylation | Publication |
| Trivedi et al.^21^ | Methylation | GEO |
| Herrera et al.^22^ | Methylation | GEO |
| Mandarano et al.^23^ | Microbiome | MECFSnet Publication |
| Nagy-Szakal et al.^24^ | Microbiome | MECFSnet Publication |
| Petty et al.^25^ | miRNA | GEO |
| Almenar-Perez et al.^26^ | miRNA | GEO |
| Nepotchatykh et al.^27^ | miRNA | Publication |
| Billing-Ross et al.^28^ | mtDNA | MECFSnet Publication |
| Milivojevic et al.^29^ | Proteomics | MECFSnet Publication |
| Raijmakers et al.^30^ | Proteomics and Microbiome | Publication |
| Germain et al.^31^ | Proteomics | MECFSnet Publication |
| Sweetman et al.^32^ | Proteomics | Publication |

*Curation is ongoing, we welcome ME/CFS research from all data sources.

**Table S2:** *Synonym Tagging performed for each data type, which is driven by including a specific column name in the Data File (‘Required Data Column(s)’) and the process used databases to tag common alternate names for molecules. With the Synonym Tagging the search space for molecules expands beyond that provided in the Data File.*

| **Data Type** | **Required Data Column(s)** | **Database used for Tagging** | **What is Searchable?** | **Example Searches** |
| --- | --- | --- | --- | --- |
| Gene Expression | - Molecule | NCBI Gene (December 2018)^33^ | - Any entry from the Molecule data column - Matching gene synonyms | - APOE - ApoE4 |
| Cytokine Assay | - Molecule | NCBI Gene (December 2018)^33^ | - Any entry from the Molecule data column - Matching gene synonyms | - IL-17 - IL-17A |
| Metabolomics | - InChiKey^34^ - Molecule - database_identifier | N/A | - User input from any of the three required columns | - QTBSBXVTEAMEQO-UHFFFAOYSA-N - Acetate - CHEBI:15366 |
| miRNA | - Molecule | miRBase (March 2019)^35^ | - Any entry from the Molecule data column - Any miRNA related to the primary transcript. - Any matching alias | - hsa-miR-198 - MIMAT0000228 |
| Methylation | - Molecule | Illumina 450K (v.15017482_v1-2) or Infinium MethylationEPIC (v-1-0-b4). | - Any entry from the Molecule data column - Corresponding B37 coordinates (Chr:Pos) | - cg12045430 - 1:29407 |

**References**

1. Giloteaux L, Goodrich JK, Walters WA, Levine SM, Ley RE, Hanson MR. Reduced diversity and altered composition of the gut microbiome in individuals with myalgic encephalomyelitis/chronic fatigue syndrome. Microbiome. 2016;4(1):30.

2. Giloteaux L, O’Neal A, Castro-Marrero J, Levine SM, Hanson MR. Cytokine profiling of extracellular vesicles isolated from plasma in myalgic encephalomyelitis/chronic fatigue syndrome: a pilot study. Journal of Translational Medicine. 2020;18(1):387.

3. Hornig M, Montoya JG, Klimas NG, Levine S, Felsenstein D, Bateman L, et al. Distinct plasma immune signatures in ME/CFS are present early in the course of illness. Science Advances. 2015;1.

4. Hornig M, Gottschalk CG, Eddy ML, Che X, Ukaigwe JE, Peterson DL, et al. Immune network analysis of cerebrospinal fluid in myalgic encephalomyelitis/chronic fatigue syndrome with atypical and classical presentations. Transl Psychiatry. 2017;7(4):e1080.

5. Montoya JG, Holmes TH, Anderson JN, Maecker HT, Rosenberg-Hasson Y, Valencia IJ, et al. Cytokine signature associated with disease severity in chronic fatigue syndrome patients. Proc Natl Acad Sci U S A. 2017;114(34):E7150-e8.

6. Bouquet J, Li T, Gardy JL, Kang X, Stevens S, Stevens J, et al. Whole blood human transcriptome and virome analysis of ME/CFS patients experiencing post-exertional malaise following cardiopulmonary exercise testing. PLoS One. 2019;14(3):e0212193.

7. Byrnes A, Jacks A, Dahlman-Wright K, Evengard B, Wright FA, Pedersen NL, et al. Gene expression in peripheral blood leukocytes in monozygotic twins discordant for chronic fatigue: no evidence of a biomarker. PLoS One. 2009;4(6):e5805.

8. Raijmakers RPH, Jansen AFM, Keijmel SP, Ter Horst R, Roerink ME, Novakovic B, et al. A possible role for mitochondrial-derived peptides humanin and MOTS-c in patients with Q fever fatigue syndrome and chronic fatigue syndrome. J Transl Med. 2019;17(1):157.

9. Gow JW, Hagan S, Herzyk P, Cannon C, Behan PO, Chaudhuri A. A gene signature for post-infectious chronic fatigue syndrome. BMC Med Genomics. 2009;2:38.

10. Armstrong CW, McGregor NR, Lewis DP, Butt HL, Gooley PR. Metabolic profiling reveals anomalous energy metabolism and oxidative stress pathways in chronic fatigue syndrome patients. Metabolomics. 2015;11(6):1626-39.

11. Germain A, Ruppert D, Levine SM, Hanson MR. Prospective Biomarkers from Plasma Metabolomics of Myalgic Encephalomyelitis/Chronic Fatigue Syndrome Implicate Redox Imbalance in Disease Symptomatology. Metabolites. 2018;8(4).

12. Germain A, Ruppert D, Levine SM, Hanson MR. Metabolic profiling of a myalgic encephalomyelitis/chronic fatigue syndrome discovery cohort reveals disturbances in fatty acid and lipid metabolism. Mol Biosyst. 2017;13(2):371-9.

13. Nagy-Szakal D, Barupal DK, Lee B, Che X, Williams BL, Kahn EJR, et al. Insights into myalgic encephalomyelitis/chronic fatigue syndrome phenotypes through comprehensive metabolomics. Sci Rep. 2018;8(1):10056.

14. Germain A, Barupal DK, Levine SM, Hanson MR. Comprehensive Circulatory Metabolomics in ME/CFS Reveals Disrupted Metabolism of Acyl Lipids and Steroids. Metabolites. 2020;10(1).

15. Kitami T, Fukuda S, Kato T, Yamaguti K, Nakatomi Y, Yamano E, et al. Deep phenotyping of myalgic encephalomyelitis/chronic fatigue syndrome in Japanese population. Scientific reports. 2020;10(1):19933-.

16. Baraniuk JN, Kern G, Narayan V, Cheema A. Exercise modifies glutamate and other metabolic biomarkers in cerebrospinal fluid from Gulf War Illness and Myalgic encephalomyelitis / Chronic Fatigue Syndrome. PLoS One. 2021;16(1):e0244116.

17. de Vega W, Erdman L, Vernon SD, Goldenberg A, McGowan PO. . Integration of DNA methylation & health scores identifies subtypes in myalgic encephalomyelitis/chronic fatigue syndrome. Epigenomics. 2018;10(5):539-57.

18. de Vega WC, Herrera S, Vernon SD, McGowan PO. Epigenetic modifications and glucocorticoid sensitivity in Myalgic Encephalomyelitis/Chronic Fatigue Syndrome (ME/CFS). BMC Med Genomics. 2017;10(1):11.

19. de Vega WC, Vernon SD, McGowan PO. DNA methylation modifications associated with chronic fatigue syndrome. PLoS One. 2014;9(8):e104757.

20. Helliwell AM, Sweetman EC, Stockwell PA, Edgar CD, Chatterjee A, Tate WP. Changes in DNA methylation profiles of myalgic encephalomyelitis/chronic fatigue syndrome patients reflect systemic dysfunctions. Clinical Epigenetics. 2020;12(1):167.

21. Trivedi MS, Oltra E, Sarria L, Rose N, Beljanski V, Fletcher MA, et al. Identification of Myalgic Encephalomyelitis/Chronic Fatigue Syndrome-associated DNA methylation patterns. PLoS One. 2018;13(7):e0201066.

22. Herrera S, de Vega WC, Ashbrook D, Vernon SD, McGowan PO. Genome-epigenome interactions associated with Myalgic Encephalomyelitis/Chronic Fatigue Syndrome. Epigenetics. 2018;13(12):1174-90.

23. Mandarano AH, Giloteaux L, Keller BA, Levine SM, Hanson MR. Eukaryotes in the gut microbiota in myalgic encephalomyelitis/chronic fatigue syndrome. PeerJ. 2018;6:e4282.

24. Nagy-Szakal D, Williams BL, Mishra N, Che X, Lee B, Bateman L, et al. Fecal metagenomic profiles in subgroups of patients with myalgic encephalomyelitis/chronic fatigue syndrome. Microbiome. 2017;5(1):44.

25. Petty RD, McCarthy NE, Le Dieu R, Kerr JR. MicroRNAs hsa-miR-99b, hsa-miR-330, hsa-miR-126 and hsa-miR-30c: Potential Diagnostic Biomarkers in Natural Killer (NK) Cells of Patients with Chronic Fatigue Syndrome (CFS)/ Myalgic Encephalomyelitis (ME). PLoS One. 2016;11(3):e0150904.

26. Almenar-Perez E, Sarria L, Nathanson L, Oltra E. Assessing diagnostic value of microRNAs from peripheral blood mononuclear cells and extracellular vesicles in Myalgic Encephalomyelitis/Chronic Fatigue Syndrome. Sci Rep. 2020;10(1):2064.

27. Nepotchatykh E, Elremaly W, Caraus I, Godbout C, Leveau C, Chalder L, et al. Profile of circulating microRNAs in myalgic encephalomyelitis and their relation to symptom severity, and disease pathophysiology. Scientific Reports. 2020;10(1):19620.

28. Billing-Ross P, Germain A, Ye K, Keinan A, Gu Z, Hanson MR. Mitochondrial DNA variants correlate with symptoms in myalgic encephalomyelitis/chronic fatigue syndrome. J Transl Med. 2016;14:19.

29. Milivojevic M, Che X, Bateman L, Cheng A, Garcia BA, Hornig M, et al. Plasma proteomic profiling suggests an association between antigen driven clonal B cell expansion and ME/CFS. PLoS One. 2020;15(7):e0236148.

30. Raijmakers RPH, Roerink ME, Jansen AFM, Keijmel SP, Gacesa R, Li Y, et al. Multi-omics examination of Q fever fatigue syndrome identifies similarities with chronic fatigue syndrome. Journal of Translational Medicine. 2020;18(1):448.

31. Germain A, Levine SM, Hanson MR. In-Depth Analysis of the Plasma Proteome in ME/CFS Exposes Disrupted Ephrin-Eph and Immune System Signaling. Proteomes. 2021;9(1).

32. Sweetman E, Kleffmann T, Edgar C, de Lange M, Vallings R, Tate W. A SWATH-MS analysis of Myalgic Encephalomyelitis/Chronic Fatigue Syndrome peripheral blood mononuclear cell proteomes reveals mitochondrial dysfunction. J Transl Med. 2020;18(1):365.

33. Maglott D, Ostell J, Pruitt KD, Tatusova T. Entrez Gene: gene-centered information at NCBI. Nucleic Acids Res. 2005;33(Database issue):D54-8.

34. Heller SR, McNaught A, Pletnev I, Stein S, Tchekhovskoi D. InChI, the IUPAC International Chemical Identifier. J Cheminform. 2015;7:23.

35. Kozomara A, Birgaoanu M, Griffiths-Jones S. miRBase: from microRNA sequences to function. Nucleic Acids Res. 2019;47(D1):D155-D62.
