# Supplementary material for: mapMECFS: a portal to enhance data discovery across biological disciplines and collaborative sites
Source: J Transl Med. 2021 Nov 8;19:461. doi: 10.1186/s12967-021-03127-3 (PMC8576927; doi:10.1186/s12967-021-03127-3)
Supplement: Supplementary file 2 — Additional file 2: Figure S2. mapMECFS expected formats for (a) data (e.g., Cytokine data), (b) phenotype, (c) results files, and (d) the summary statistics file format. [file 12967_2021_3127_MOESM2_ESM.pdf]

(a)

Cytokine Data File Format
Tab-separated file

Column 1: Cytokine name. Column name must be 'Molecule'. These must be UNIQUE and cannot be blank ('').

2nd column and onwards are the participant IDs. These must be UNIQUE and match the phenotype file.

Each column contains measurements from 1 sample. Missing values are allowed ('NA' or leave empty).

Molecule	MMC000001	MMC000002	MMC000003	MMC000004	MMC000005	MMC000006
sCD40L	323.7634	358.5415	310.0712	287.3893	310.4637	272.7896
EGF	49.41162	52.74591	53.72772	52.69184	55.8378	50.60854

(b)

Phenotype File Format
Tab-separated file

Column 1: ID for each participant. Column header must be 'ParticipantID'. Column is REQUIRED. These must be UNIQUE and match the data file.

Column 2: Phenotype of interest. Column header must be 'Phenotype'. Column is REQUIRED.

Column 3: The source or tissue the sample was extracted from. Column header must be 'Sample_Source'. Column is required. Missing is allowed ('NA' or empty string).

Columns are optional. You can substitute with any other information.

Each column contains data for 1 participant. Missing values are allowed ('NA' or leave empty).

ParticipantID	Phenotype	Sample_Source	characteristics_ch1.0.twin pair	characteristics_ch1.1.sex
GSM402241	unaffected	PBLs	228340	female
GSM402242	CFS	PBLs	228340	female

(c)

Results File Format
Tab-separated file

Column 1: ID for the molecule measured (transcript IDs in this example). Column name must be 'Molecule'. This is REQUIRED.

Column 2: Other OPTIONAL Identifiers.

Column 3 and 4: Number of cases ('NCases') and controls ('NControls') for each molecule. Use these columns for analyses where this varies by molecule. OPTIONAL

Optional Columns: Various analysis results. Columns are flexible to the analysis conducted.

Each column contains data for 1 molecule. Missing values are allowed ('NA' or leave empty).

Pvalue: p-value of the test. Column name must be 'Pvalue'.
PvalueAdj: adjusted p-value for the test. Column name must be 'PvalueAdj'.

Molecule	Gene	NCases	NControls	baseMean	log2FoldChange	lfcSE	stat	Pvalue	PvalueAdj
NM_000014	A2M	100	100	1889.680119	-0.46319172	0.52404	-0.4848	0.80035	0.984757
NM_000015	NAT2	98	99	[illegible]	[illegible]	[illegible]	[illegible]	[illegible]	[illegible]

(d)

Summary Statistics File Format

The summary statistics file is generated automatically by mapMECFS. It is available for download as a tab-separated file.

Column 1: ID for molecule in the dataset.

Column 2: The source or tissue the sample was extracted from. Same as phenotype file.

Columns 3-4: Sample Size

Columns 5-6: Median

Columns 7-8: Standard Deviation

Column 9: Wilcoxon rank-sum statistic

Column 10: Wilcoxon rank-sum p-value

Column 11: Wilcoxon rank-sum Bonferroni corrected p-value

Molecule	Sample_Source	count | HC	count | ME/CFS	median | HC	median | ME/CFS	std | HC	std | ME/CFS	Ranksum stat | ME/CFS/HC	Ranksum p-value | ME/CFS/HC	Ranksum bonf | ME/CFS/HC
cg00000029	PBMC	12	13	5.94E-01	5.63E-01	4.50E-02	3.99E-02	-2.3389	1.93E-02	1
cg00000155	PBMC	12	13	9.69E-01	9.69E-01	1.14E-02	7.75E-03	-1.63E-01	8.70E-01	1
cg00000158	PBMC	12	13	[illegible]	[illegible]	[illegible]	[illegible]	[illegible]	[illegible]	1

Additional File 2: Figure S2: mapMECFS expected formats for (a) data (e.g. Cytokine data), (b) phenotype, (c) results files, and (d) the summary statistics file format. All of these helper information is available at mapMECFS about page (https://www.mapmecfs.org/about).
