# Supplementary figures and images for: mapMECFS: a portal to enhance data discovery across biological disciplines and collaborative sites
Source: J Transl Med. 2021 Nov 8;19:461. doi: 10.1186/s12967-021-03127-3 (PMC8576927; doi:10.1186/s12967-021-03127-3)

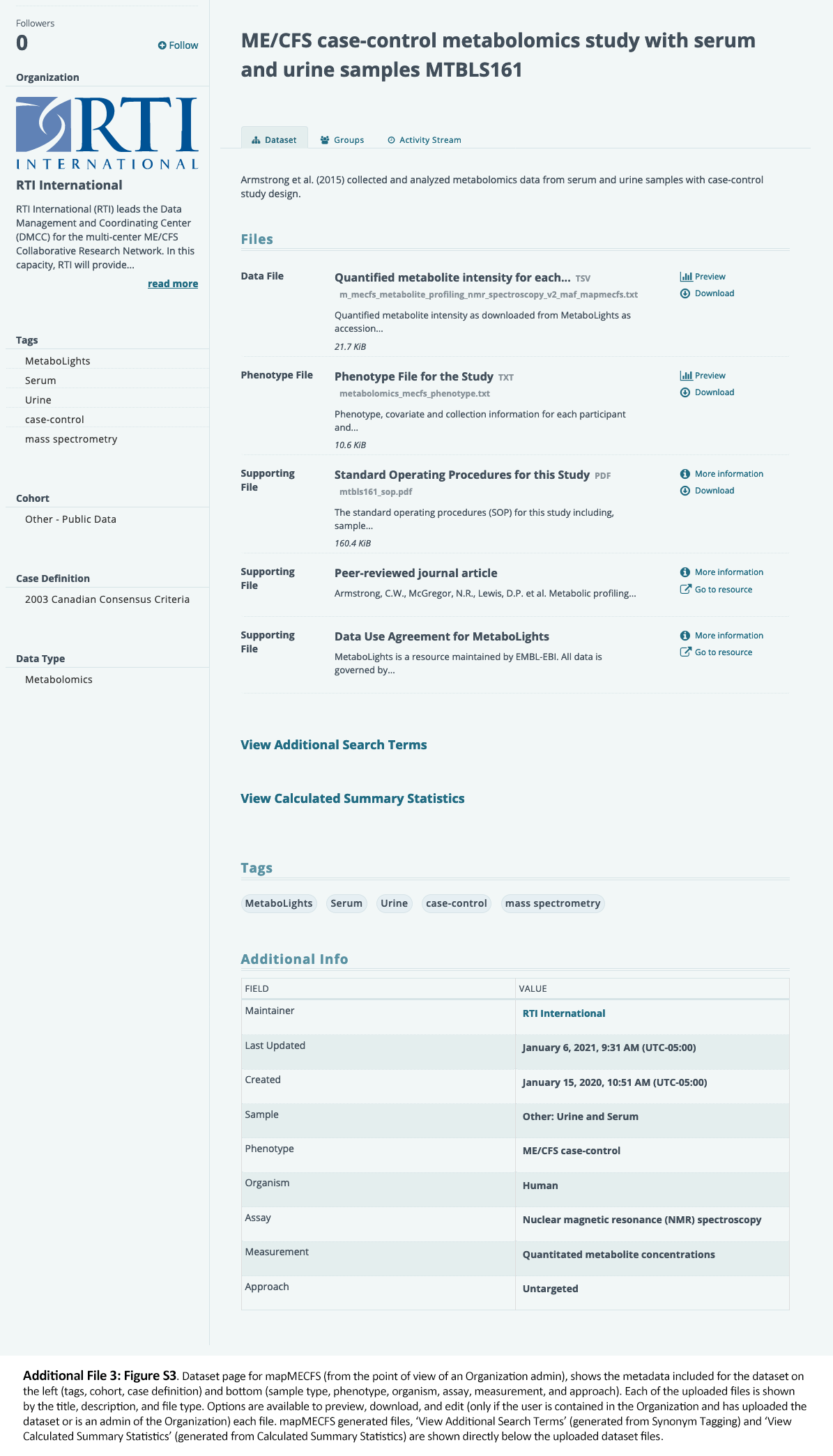

Supplement: Supplementary file 3 — Additional file 3: Figure S3. Dataset page for mapMECFS shows the metadata included for the dataset on the left. Each of the uploaded files is shown by the title, description, and file type. Options are available to preview, download, and edit each file. mapMECFS generated files are shown directly below the uploaded dataset files. [file 12967_2021_3127_MOESM3_ESM.png]

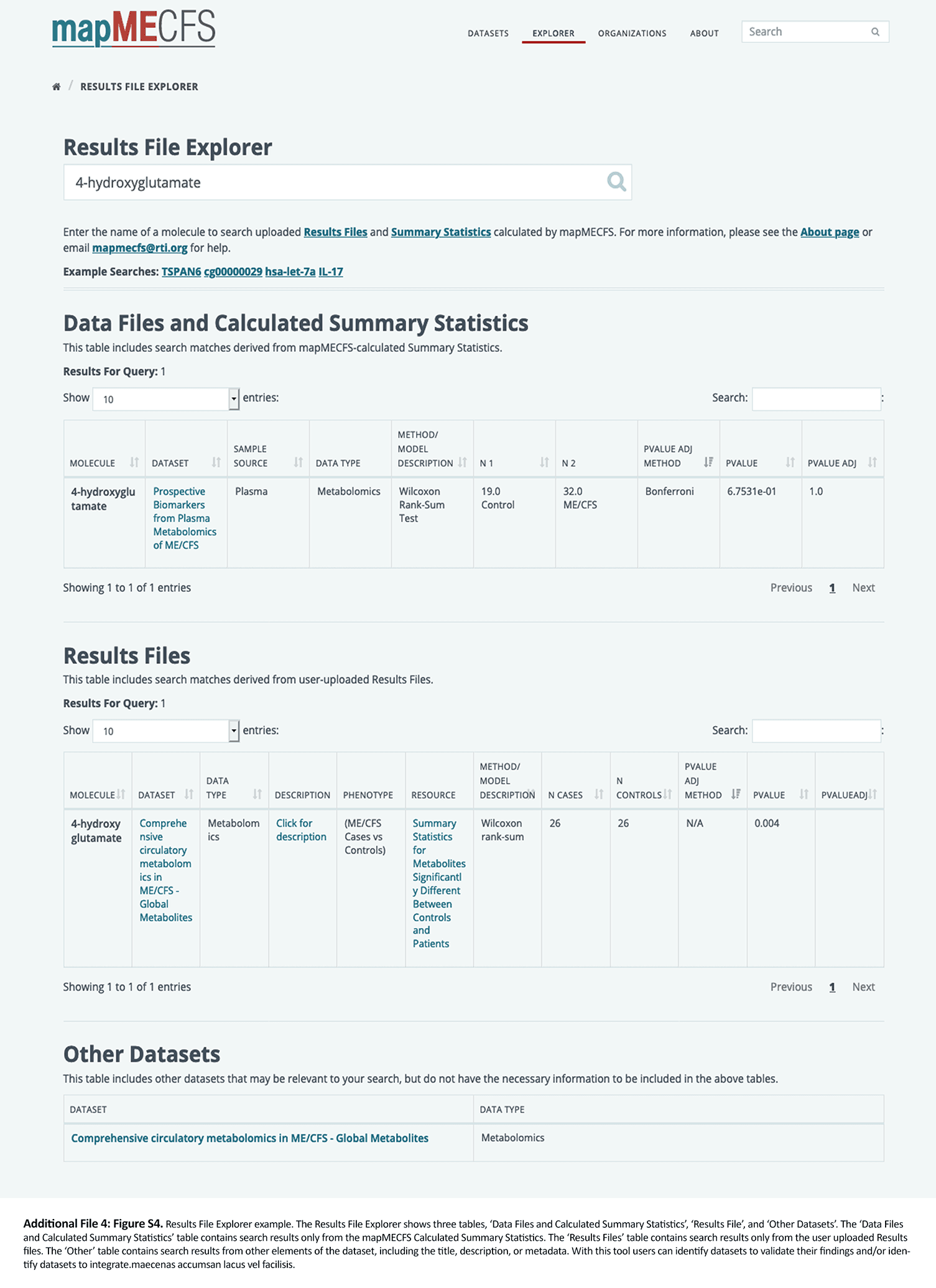

Supplement: Supplementary file 4 — Additional file 4: Figure S4. Description: Results File Explorer example with a search for 4-hydroxyglutamate. The Results File Explorer shows three tables, “Data Files” and “Calculated Summary States,” “Results File,” and “Other Datasets.” [file 12967_2021_3127_MOESM4_ESM.png]
